# Supplementary material for: Therapeutic role of recurrent ESR1-CCDC170 gene fusions in breast cancer endocrine resistance
Source: Breast Cancer Res. 2020 Aug 8;22:84. doi: 10.1186/s13058-020-01325-3 (PMC7414578; doi:10.1186/s13058-020-01325-3)
Supplement: Supplementary file 2 — Additional file 2: Figure S2. Verifying the ectopic expression of ESR1-CCDC170 fusion protein products in engineered T47D cells lines by Western Blots. [file 13058_2020_1325_MOESM2_ESM.pptx]

## Slide 1
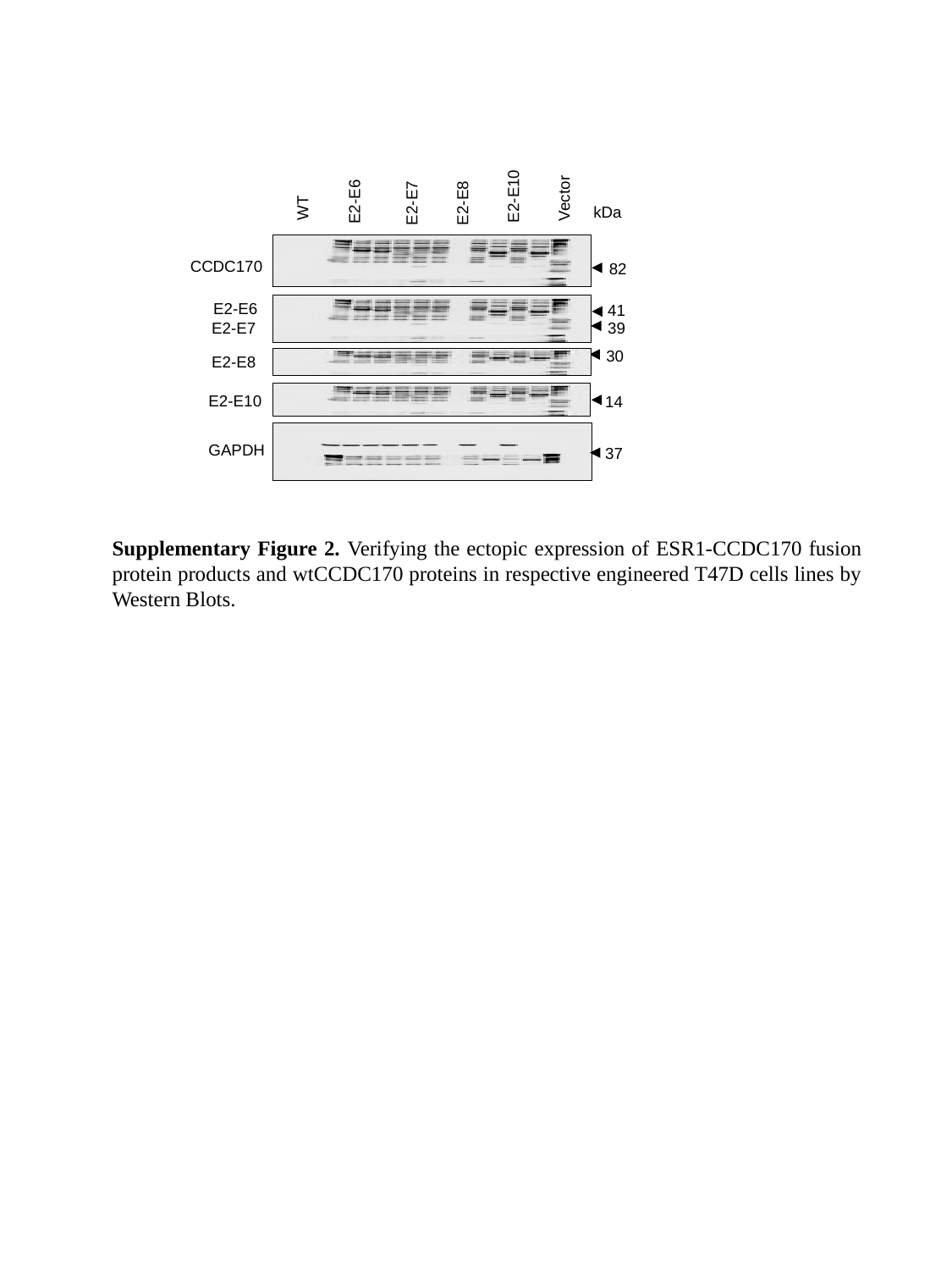

E2-E10
Vector
E2-E6
E2-E7
E2-E8
WT
CCDC170
E2-E6
E2-E7
E2-E8
E2-E10
GAPDH
kDa
82
41
39
30
14
37
Supplementary Figure 2. Verifying the ectopic expression of ESR1-CCDC170 fusion protein products and wtCCDC170 proteins in respective engineered T47D cells lines by Western Blots.
