# Supplementary material for: Therapeutic role of recurrent ESR1-CCDC170 gene fusions in breast cancer endocrine resistance
Source: Breast Cancer Res. 2020 Aug 8;22:84. doi: 10.1186/s13058-020-01325-3 (PMC7414578; doi:10.1186/s13058-020-01325-3)
Supplement: Supplementary file 5 — Additional file 5: Figure S5. Ectopically expressed V5-tagged ΔCCDC170 co-precipitates with HER2. [file 13058_2020_1325_MOESM5_ESM.pptx]

## Slide 1
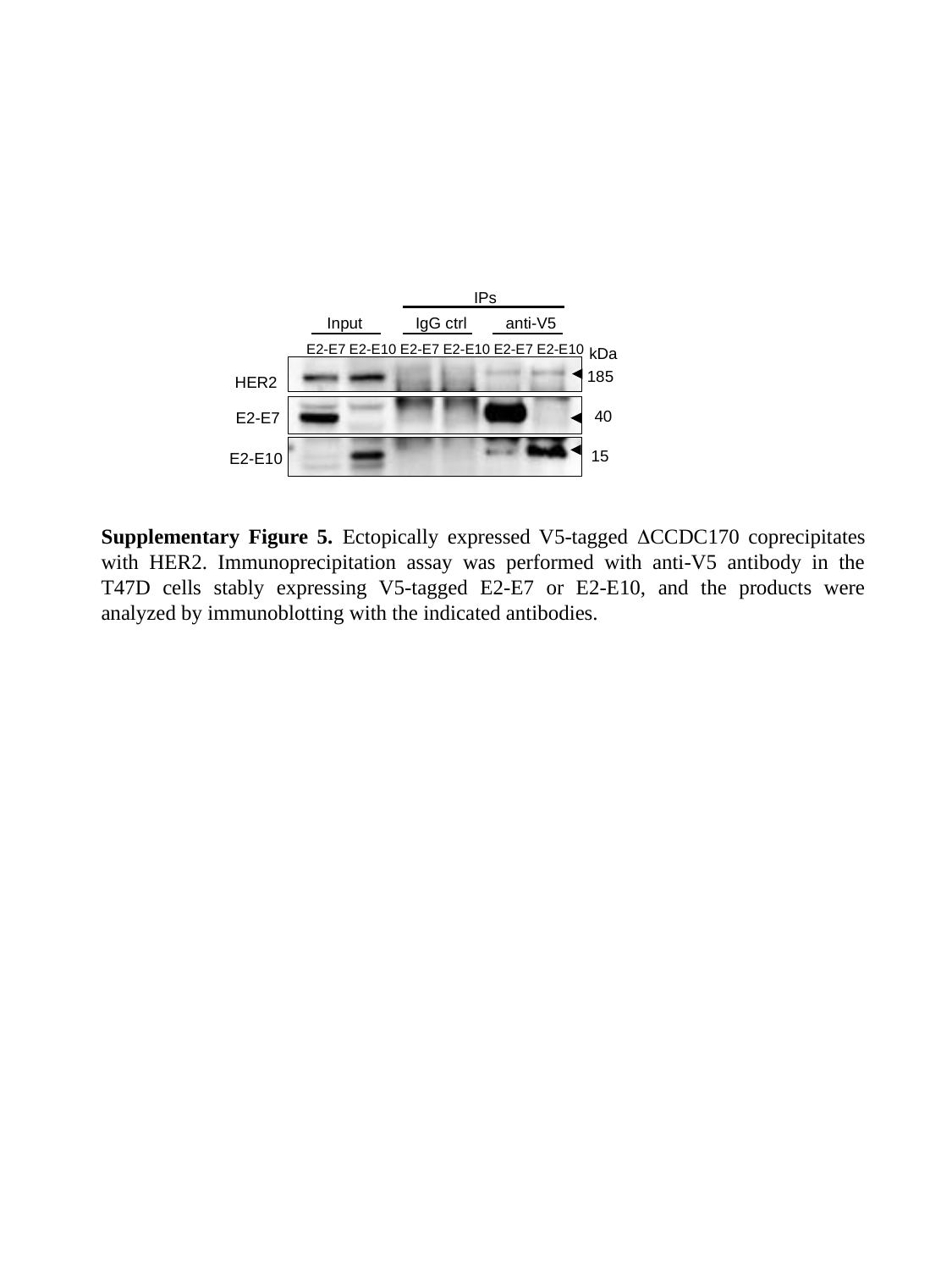

IPs
Input
IgG ctrl
anti-V5
E2-E7
E2-E10
E2-E7
E2-E10
E2-E7
E2-E10
HER2
E2-E7
E2-E10
185
40
15
kDa
Supplementary Figure 5. Ectopically expressed V5-tagged ΔCCDC170 coprecipitates with HER2. Immunoprecipitation assay was performed with anti-V5 antibody in the T47D cells stably expressing V5-tagged E2-E7 or E2-E10, and the products were analyzed by immunoblotting with the indicated antibodies.
