# Supplementary material for: Therapeutic role of recurrent ESR1-CCDC170 gene fusions in breast cancer endocrine resistance
Source: Breast Cancer Res. 2020 Aug 8;22:84. doi: 10.1186/s13058-020-01325-3 (PMC7414578; doi:10.1186/s13058-020-01325-3)
Supplement: Supplementary file 9 — Additional file 9: Figure S9. Western blots detecting HER2, HER3, and SRC protein expression in the cell models used in this study. [file 13058_2020_1325_MOESM9_ESM.pptx]

## Slide 1
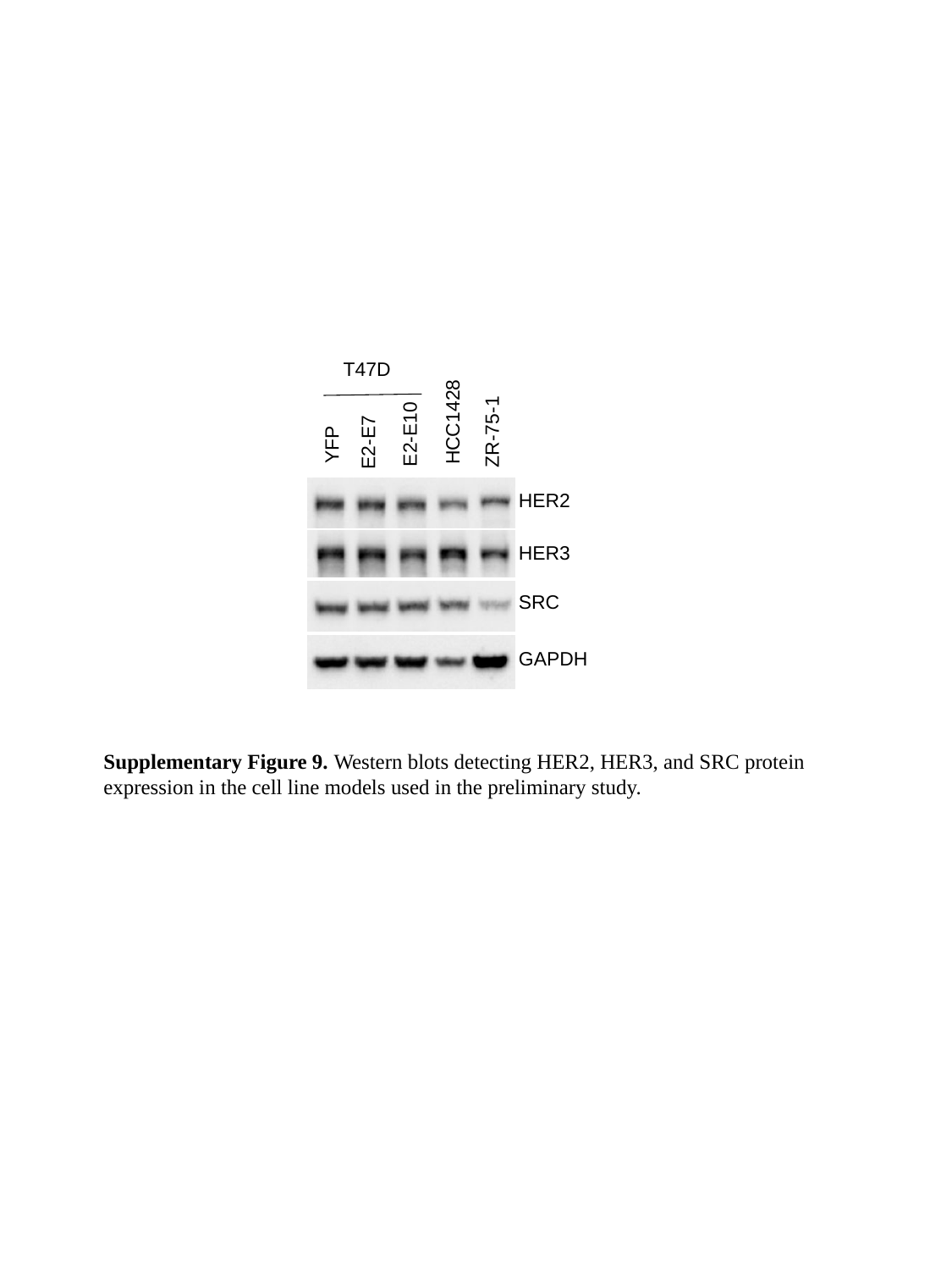

T47D
HCC1428
ZR-75-1
E2-E10
E2-E7
YFP
HER2
HER3
SRC
GAPDH
Supplementary Figure 9. Western blots detecting HER2, HER3, and SRC protein expression in the cell line models used in the preliminary study.
